# Perceptions of Adults Aged 50 Years and Older Regarding the Use of Wearable mHealth Technologies to Promote Physical Activity: Systematic Review and Meta-Ethnography

**DOI:** 10.2196/67157

**Published:** 2026-07-29

**Authors:** Beatriz Rodríguez-Martín, Diego González-Arroyo, Susana Priego-Jiménez, María Lopez-González, María Medrano-Echeverría, Marta Carolina Ruiz-Grao, Celia Álvarez-Bueno

**Affiliations:** 1 Department of Nursing, Faculty of Health Sciences, Physiotherapy and Occupational Therapy, University of Castilla-La Mancha, Talavera de la Reina, Spain; 2 ABC-Age Research Group, University of Castilla-La Mancha, Cuenca, Spain; 3 Network for Research on Chronicity, Primary Care, and Health Promotion (RICAPPS), Barcelona, Spain; 4 University Hospital Nuestra Señora del Prado, Health Service of Castilla-La Mancha, Talavera de la Reina, Spain; 5 University Hospital of Cuenca, Camino de El Terminillo s/n, Cuenca, 16003, Spain, 34 969 17 99 00; 6 Department of Health Sciences, Institute for Innovation & Sustainable Food Chain Development, Public University of Navarra, Pamplona, Spain; 7 CIBER de Fisiopatología de la Obesidad y Nutrición (CIBEROBN), Instituto de Salud Carlos III, Madrid, Spain; 8 Department of Nursing, Nursing Faculty, Physiotherapy and Occupational Therapy, University of Castilla-La Mancha. Albacete, Albacete, Spain; 9 Universidad Politécnica y Artística del Paraguay, Asuncion, Paraguay

**Keywords:** eHealth, mHealth, mobile health, mobile app, app, application, smartphone, digital health, digital technology, digital intervention, electronic devices, meta-ethnography, older people, qualitative, physical activity, physical exercise, exercise, exercising, systematic review, review

## Abstract

**Background:**

Despite advances in wearable mobile health (mHealth) technologies and their associated apps designed to promote physical activity, and the importance of adapting them to users, little is known about older adults’ perceptions of these technologies.

**Objective:**

This review aimed to synthesize and analyze qualitative evidence exploring the perceptions of adults aged 50 years and older regarding areas to improve, barriers to, and facilitators of wearable mHealth technologies (activity trackers and companion apps) to promote physical activity.

**Methods:**

A qualitative systematic review and meta-ethnography was conducted. Comprehensive searches were performed across 8 databases (MEDLINE, Scopus, Web of Science, CINAHL, The Cochrane Library Plus, PsycINFO, ProQuest, and ÍnDICEs-CSIC) for articles published in English or Spanish between January 2013 and January 2024. The synthesis followed the PRISMA (Preferred Reporting Items for Systematic Reviews and Meta-Analyses) and ENTREQ (Enhancing Transparency in Reporting the Synthesis of Qualitative Research) guidelines.

**Results:**

Ten articles met the inclusion criteria and were synthesized using meta-ethnography. Three main themes emerged: (1) barriers to promoting physical activity caused by wearable mHealth technologies: personal barriers (physical aspects, perceptions about technology, and personal preferences), technological barriers (functionality, content, design, alarms, availability, and accessibility), and environmental barriers (season of the year); (2) personal facilitators (consideration that these apps improve health, perceptions about technology, and personal preferences), technological facilitators (functionality, content, and design), relational facilitators (technological and social support), environmental facilitators (seasonal variations), and health care professionals (support and monitoring by health care services); and (3) personal areas (perceptions about technology and personal preferences), technological areas (functionality, content, and design), and relational areas (technological support).

**Conclusions:**

Although older adults acknowledge the potential of wearable mHealth technologies to promote physical activity, their effective engagement is hindered by distinct personal, technological, and environmental barriers. Bridging the digital divide requires designers to prioritize user-centered, age-friendly interfaces that are integrated with continuous support from health care professionals. To promote genuine health equity, future research must rigorously report intersectional demographics to ensure that mHealth interventions mitigate, rather than inadvertently exacerbate, existing disparities.

## Introduction

Population aging is a phenomenon of increasing magnitude worldwide. By 2025, approximately 1.2 billion people will be aged 60 years and older, reaching 2 billion by 2050 [1]. Additionally, according to the World Health Organization (WHO) forecasts, between 2000 and 2050, the percentage of people aged 60 years and older will double from 11% to 22% of the global population [2-5], surpassing the remaining population groups [6].

Population aging is associated with an increase in preventable chronic pathologies, such as rheumatism, arterial hypertension, heart disease, diabetes, chronic obstructive pulmonary disease, cerebrovascular disease, hearing loss, cataracts, depression, and dementia, as well as increasing disability, dependence, and the use of health system resources [7], which increases starting at age 55 years in men and from 60 years or more in women [7,8], accounting for 70%‐80% of health expenditures in Europe [9]. Older adults consume 50% of the time spent with primary care professionals, 70% of the time of geriatricians, and 62% of the time spent on pharmaceutical expenditure [7], which can be reduced by half if aging is accompanied by good health, reinforcing primary care and senior services [9].

Physical activity (PA), defined as any movement that involves energy consumption, has been shown to have physical and mental benefits for older adults, such as reducing cardiovascular disease, increasing quality of life, and maintaining independence [10-12]. The WHO recommends that adults older than 65 years of age should perform between 150 and 300 minutes of moderate-intensity aerobic PA or between 75 and 150 minutes of vigorous-intensity PA per week. In addition, performing 2 or more days of muscle-strengthening activities for all muscle groups and performing PA 3 or more days a week are recommended, prioritizing functional balance and moderate-intensity strength [13]. The benefits of these recommendations include decreasing the incidence of hypertension, cancer, and type 2 diabetes; improving mental health, cognitive health, and sleep; reducing cardiovascular mortality; preventing falls; and worsening bone and functional health [2,10,13,14]. Therefore, effective interventions aimed at promoting PA among older adults are necessary [14].

Information and communication technologies in health, which are being increasingly used by older people, have revolutionized how health services address the challenges of improving the quality, effectiveness, and safety of care [15,16]. Such technologies are great tools for promoting active aging. Prominent among these technologies is eHealth, defined by the WHO as “the cost-effective and safe use of information and communication technologies in support of health-related areas, including health services, health surveillance, health documentation and education, health knowledge and research” [17]. Within eHealth is mHealth, defined as medical and public health practices supported by mobile devices. In the context of PA promotion, mHealth rarely operates as a stand-alone software app; rather, it functions as a broader, integrated ecosystem that combines hardware (such as wearable activity trackers, smartbands, or smartwatches) and companion software apps [18]. The inclusion of these hybrid interventions is crucial, as their physical nature (hardware) and digital interface (software) jointly increase older adults’ self-care, autonomy in decision-making, and self-efficacy in disease management. Furthermore, mHealth motivates older adults to engage in PA. At the systemic level, these technologies can improve care quality, reduce health care costs, and prevent unnecessary medical consultations. For the users, mHealth facilitates social interaction, digital inclusion, and intergenerational relationships, ultimately reducing loneliness and enhancing overall quality of life [19,20]. To this end, the characteristics of older adults, the ease and intuition of the use of devices and apps, their accuracy and veracity, and their needs, desires, and limitations should be considered [21-23]. Despite the benefits of these technologies for older people, factors such as anxiety and fear of technology, deficits in cognitive skills, health problems, lack of instructions and guidelines, and the cost of the technologies are barriers to their use [19]. Perceptions about the use of mHealth devices and apps to promote PA have been studied among parents and adolescents, which has revealed their satisfaction with these technologies and their effectiveness in promoting PA among children and adolescents [24]; however, few qualitative studies have analyzed their benefits among older adults, and few existing studies have focused on populations with specific pathologies, such as myeloid neoplasms [25] or breast cancer [26].

The success of such interventions depends on their ability to adapt to the needs of the user; thus, understanding how older adults can influence the use of mHealth devices and apps for the promotion of PA and analyzing their demands, contexts, and desires to develop more accessible, age-friendly, and acceptable technologies for this population group are essential [19].

This systematic review of qualitative evidence aimed to synthesize and analyze studies that explore the perceptions of adults aged 50 years and older regarding areas to improve, barriers, and facilitators of wearable mHealth technologies to promote PA. This information is key both for the tech developers and for the health care professionals who recommend, train, and supervise users in their use.

## Methods

### Overview

A qualitative systematic review and meta-ethnography were carried out. Noblit and Hare [27] described this type of synthesis of qualitative research results, which is the most widely used qualitative synthesis approach in health care. The protocol of this review was registered in PROSPERO (CDR498374). This review was conducted following the recommendations of the Cochrane Qualitative and Implementation Methods Group Guidance [28], the ENTREQ (Enhancing Transparency in Reporting the Synthesis of Qualitative Research) statement, and the criteria of the PRISMA (Preferred Reporting Items for Systematic Reviews and Meta-Analyses) Statement [29] (Checklist 1).

### Eligibility Criteria

The following inclusion and exclusion criteria were used to select the articles.

The inclusion criteria were as follows: (1) published in English and Spanish from January 2013 to January 2024 to include the latest publications on the subject, (2) used any type of qualitative approach or qualitative data collection technique, and (3) analyzed the use of mHealth ecosystems, specifically wearable PA trackers and their associated mobile apps, for the promotion of PA in healthy or chronically ill adults aged 50 years and older who were community-dwelling or institutionalized, functionally autonomous, and not classified as frail. The exclusion criteria were as follows: (1) studies that included participants with cognitive impairment or any mental pathology, (2) protocol studies or studies not completed, (3) mixed designs if the qualitative data were not analyzed in a disaggregated manner, and (4) studies that included the use of the wearable mHealth technologies exclusively for dietary recommendations.

### Data Sources and Search Strategy

Two researchers independently conducted the search and selection of articles and resolved discrepancies through consensus, with a third researcher consulted when necessary, following the established criteria in the following databases: MEDLINE (PubMed), Scopus, Web of Science, ÍnDICEs-CSIC, ProQuest, PsycINFO, The Cochrane Library Plus, and CINAHL. In addition, a secondary search was conducted using the reference lists of the selected articles. In cases of disagreement, a third researcher approved the study. The search strategy included a combination of keywords adapted to each database. The complete search strategy is presented in Multimedia Appendix 1.

### Study Selection and Data Abstraction

Article selection was performed independently by 2 researchers, with disagreements resolved by a third reviewer. First, the titles and abstracts of the articles were examined, and those that did not meet the inclusion criteria were discarded. The full texts of the selected articles were subsequently read, and the following information was extracted from each of them using an ad hoc Excel template: authors, year of publication, paradigmatic approach, objectives of the study, characteristics of the sample and sampling techniques, data collection techniques, main results, conclusions, and evaluation of methodological quality.

### Assessment of Methodological Quality

The quality of the studies was assessed with the Joanna Briggs Institute Qualitative Assessment and Review Instrument (JBI-QARI) [30], which includes 10 items with 3 response options, “yes,” “no,” and “unclear,” to evaluate the methodological quality of the qualitative studies. Studies with poor methodological quality (fewer than 5 items) were rated as “excluded” and removed from the review.

### Data Analysis and Synthesis

Following Noblit and Hare’s interpretive approach [27], the findings were synthesized through reciprocal translation, evolving into a line of argument that provides an original theoretical reconceptualization of mHealth apps to promote PA.

Data were systematically categorized using an abstraction form designed to distinguish between first-order constructs (participants’ quotations and metaphors), second-order constructs (interpretations based on patients’ quotations made by the original researchers), and third-order constructs (interpretations generated by our research team). By juxtaposing these layers, we moved beyond data aggregation toward a reciprocal translation of findings, synthesizing these accounts into a novel line of argument that reconceptualizes the phenomenon within the current literature (Multimedia Appendix 2).

The main themes, categories, subcategories, and codes were extracted. The codes were grouped by areas of similarity, resulting in the different themes, categories, and subcategories of facilitators, barriers, and areas for improvement for the promotion of PA among older adults. For the translation of the studies, the data were exported to a table containing the thematic tree, categories, subcategories, codes, and quotations to help us understand the relationships between the studies. No refutations or contradictions were found, as the data from all included studies were very similar. Finally, the researchers compared the translations to identify the lines of argument from which the synthesis would be developed.

### Research Team and Reflexivity

The research team has previous experience in qualitative research, evidence synthesis, PA, and research involving older people. The team included registered nurses, physiotherapists, an anthropologist, and a graduate in PA. The findings were analyzed from an interprofessional perspective.

## Results

### Study Selection and Characteristics

**Figure 1.**
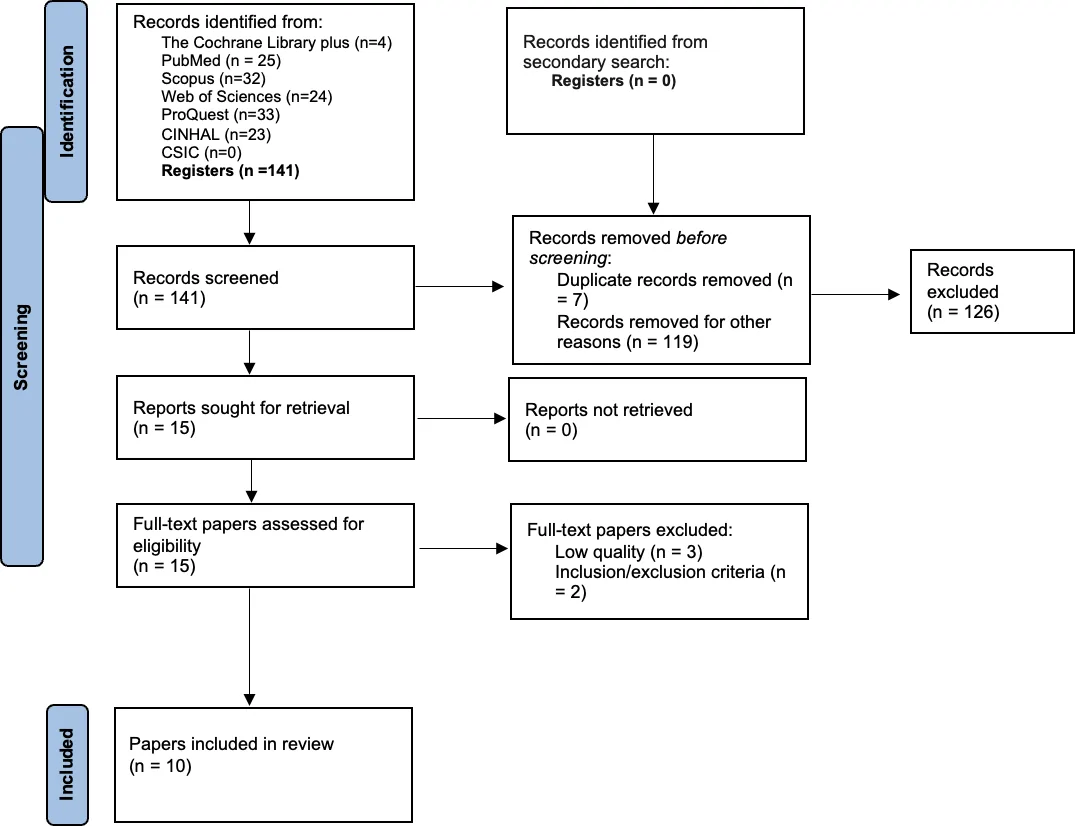
Flowchart of the search and selection process.

Following the database search, 141 references were identified, 7 of which were eliminated because they were duplicates across several databases. After the title and abstract were read, 15 articles met the inclusion criteria; 5 were excluded because of low methodological quality, specifically the use of the Joanna Briggs Institute tool for qualitative studies. Finally, 10 articles were included in the narrative synthesis and meta-ethnography (Figure 1).

### Synthesized Findings

The main characteristics of the studies analyzed are summarized in Table 1.

**Table 1.** Main characteristics of the studies analyzed.

Author and year	Paradigmatic approach	Objectives of the study	Sample characteristics and sampling technique	Data collection technique	Main results	Conclusions	Methodological quality
Moore et al (2021) [31]	Qualitative systematic review, meta-ethnography type	To synthesize qualitative studies that analyzed the experience of older people who had used a PA^a^ monitoring device for several days, and to determine which factors contributed to the acceptance and use of portable devices	Twenty articles were included with a total of 349 participants aged 51 to 94 years who had used portable devices for several days and had analyzed their experiences Intentional convenience sampling technique	Semistructured interviews and focus groups	Three main themes explained older adults’ experience with physical activity monitoring devices Barriers: age-associated physiology and comorbidities. They were frustrated when the devices did not have the functions they wanted, were difficult to access, did not achieve their goal or capture all their activities, and had to rely on family or friends; when they were inaccurate, cumbersome, difficult to connect, incompatible with their phone, and complex instructions Facilitators: continuous monitoring of the device and reaching their proposed goal. Supported by their family, friends, and health care personnel. That the devices were waterproof, small, comfortable, easy to put on and handle, safe, pleasant and easy to read display, long battery life, with a thin and flexible strap; that counted steps and had GPS, could measure health parameters, inform them of their progress and automatically synchronize with their other devices Areas for improvement: clear and simple instructions, that the device was acceptable and reliable and that they did not have to charge it constantly	The factors that positively influence the use of PA devices and apps in older adults are personal motivation, ease of use, the objective set by the users, and considering their preferences. However, to overcome perceived barriers, such as usability or physiology-related barriers, older adults must be motivated and socially supported. In addition, device-related features (charging, instructions, and so on) should be improved.	10/10
Peng et al (2021) [32]	Phenomenology	To learn how users develop long-term habits following the use of PA monitoring devices (PA trackers)	20 adults over 65 years of age (11 women and 9 men) who had worn activity trackers for more than 6 months Intentional convenience sampling technique	Two semistructured interviews were conducted with each participant	Analysis of the data obtained revealed the participants' perceptions of the use of physical activity trackers, classified into the following facilitators: Facilitators: age-associated comorbidities. Setting a small goal and increasing it. Use of reminders and routines: they wore them first thing in the morning, charged them during the night, and always left them in the same place; they also used them in situations such as TV commercials, telephone conversations, or explored new routes to achieve the set goal. They were able to anticipate potential obstacles that might prevent them from using the activity tracker (eg, running out of battery). In addition, they were prepared for dissatisfaction with not achieving the set goal and occasional lapses with a positive mindset and attitude	Contextual factors (such as the place where they place the tracker) and technological factors (such as set goals, reminders...) facilitate the adherence of older adults to use PA monitoring devices in the long term. These user-identified factors can be used to design next-generation wearable activity trackers and facilitate behavior change in older adults.	10/10
Kononova et al (2019) [33]	Phenomenology	To determine the factors that favored or did not favor the use of activity trackers in people aged 65 years and older	48 people aged 65 years and older, of whom 17 had not used trackers, 9 had been using them in the short term, 11 had stopped using them, and 11 had used them in the long term Intentional convenience sampling technique	Ten focus groups	Three themes explained participants' perceptions of the use of physical activity trackers that were categorized into the following barriers, facilitators, and areas for improvement: Barriers: the devices did not have the function of establishing routines or an exercise schedule; inaccuracy in step counting, high price of the device, low interest in the device, and fear of sharing their data Facilitators: that the device was attractive, with a comfortable bracelet and easy-to-read screen, waterproof, with long battery life; that other parameters such as sleep, heart rate, and so on, could be monitored; that the device allowed them to be more aware of their daily activity since its use reduced stress and promoted their independence; and to set goals, thus competing with family and friends Areas for improvement: instructions for use and training on functionality	Improving the features and functions of mhealth^b^ devices and apps perceived as barriers by older adults are essential aspects for their use. However, being aware of the long-term benefits of app use, perceived social support, and motivation are facilitators that favor older adults' adherence to these devices.	10/10
Abouzahra and Ghasemaghaei (2020) [34]	Phenomenology	To examine the influence and effect of the use of PA monitoring devices in older adults	44 persons aged 65 years and older (20 men and 24 women) who had never used any PA monitoring device. Intentional convenience sampling technique.	Two semistructured interviews with each participant.	The analysis of the data revealed 3 themes, classified into the following barriers, facilitators, and areas for improvement in the use of PA monitoring devices in older adults: Barriers: inexperience in the use of technologies and the complexity of the device. The use of alarms and the high price of the device Facilitators: alarms, previous experience with other technologies, and support from friends and family Areas for improvement: monitoring other health parameters	The use of PA monitoring devices in older adults is a complex process influenced by social (support from family and friends...), psychological (problem-solving skills), and technological (device complexity) factors. Taking these factors into account, strategies can be developed to improve the use of portable devices in this population and improve their health and quality of life.	10/10
Ehn et al (2018) [35]	Phenomenology	To explore the experiences, requirements, and preferences of older adults for the use of PA monitoring devices	Eight older adults aged 75 years and older; 6 had a mild walking disability and used wheeled walkers, and 2 participants walked without assistance. All knew how to use a smartphone, had not used an iPad, and 2 of them used computers. Two physical activity recording wristbands were used. Intentional convenience sampling technique.	Individual semistructured interviews and focus groups.	Three themes explained the participants' experiences using the activity monitoring devices that were categorized into barriers, facilitators, and areas for improvement: Barriers: inaccurate measurements made by the devices. Many perceived the devices as fragile and therefore could break or damage them. They were afraid of being monitored and losing privacy, and some stopped using the device when it caused some kind of problem. Another barrier was that the device instructions were not in their native language. Facilitators: achieving the proposed goal by making them more aware of how active they were even indoors. The use of reminders and previous experience using other technologies made it easier for them to use the devices. They preferred the wristbands to be waterproof, flexible, small, comfortable, and easy to combine with clothing. Areas for improvement: combining PA measurements with other health parameters and improvements in device battery life	PA monitoring devices are an important tool to promote physical activity in older adults, but these should be easy to use and intuitive, with accurate activity log measurements, and display motivational messages.	10/10
Fausset et al (2013) [36]	Phenomenology	To explore the use and attitudes of older adults toward 4 PA monitoring technologies and to learn how they integrate these devices into their daily lives	Four married couples (6 perceived their health status as optimal and 2 as fair or poor) had a computer and internet connection and had no previous experience with any activity monitoring technology. Intentional convenience sampling technique	Two semistructured interviews with each participant.	The most significant findings of the study were classified into barriers, facilitators, and areas for improvement. Barriers: the inaccuracy in measuring the parameters, the high price, and the discomfort of wearing the bracelet at night Facilitators: the enthusiasm and willingness shown by the participants to use the device and to be able to count the steps taken correctly. Areas for improvement: long-term use, since half of the participants stated that they would not use the device continuously	Older adults perceive PA monitoring devices to be easy to use and useful as a key instrument for the promotion of healthy lifestyles. However, their high price, the inaccuracy of data measurement, and the loss of time in connecting and putting on the device are barriers to the use of these devices.	10/10
Mercer et al (2016) [37]	Phenomenology	To examine the usability and usefulness of activity monitoring devices in chronically ill older adults	32 participants between 52 and 84 years of age with a chronic pathology. Twelve participants did not have a cell phone, so they were provided with one. The participants previously used a pedometer. Subsequently, they were assigned a device to use for at least 3 days. Intentional convenience sampling technique	Two focus groups were conducted.	Three themes emerged in the study that described the acceptability of the devices by the older adults, which were classified into barriers, facilitators, and areas for improvement: Barriers: participants were uncomfortable using the device because they thought they might break it. Facilitators: that the devices were easy to use and made them more aware of their PA level, thanks to the alerts function, turning the set daily step goal into a game or competition with other users or with themselves. Finally, participants reflected that these devices could be useful in the health sector to improve their health and that they should be available in pharmacies and tax-free. Areas for improvement: a simple manual of instructions, less technical terms, and clearer screens.	For older adults with chronic diseases, wearable physical activity trackers are useful and acceptable. However, these users may need help setting up the device and learning how to interpret the data obtained.	10/10
Puri et al (2017) [38]	Mixed design, longitudinal quantitative study and qualitative study based on phenomenology	To evaluate the acceptability and use of activity monitoring devices in older adults considering the influencing factors identified in the literature and the technology acceptance model	20 adults older than 55 years of age, 12 women and 8 men. Of the total, 18 used a computer daily, 14 owned a personal smartphone, and 17 had heard of activity monitoring devices. Intentional convenience sampling technique	Four semistructured interviews were conducted in the qualitative part.	Three themes explained older adults' perceptions of the acceptability of activity monitoring devices organized into the following barriers, facilitators, and areas for improvement. Barriers: participants' lack of prior experience and seniors' sense of independence caused participants not to seek support from loved ones. Facilitators: battery life, thin and flexible wristbands, sharing data with other users, and quick access to information managed by the device to understand the level and intensity of PA performed. In addition, the increased self-awareness of the device motivated them to make lifestyle changes and to inform themselves about health-related topics. Areas for improvement: the devices had to be easy to use, comfortable, and they were not afraid of breaking them	Most older adults accept PA monitoring devices and perceive them as valuable to their lives. In addition, they are concerned about the features of the trackers (comfort, aesthetics, and price), whereas privacy is less of a concern for this group	10/10
Schlomann et al (2016) [39]	Phenomenology	To assess the experiences, opportunities, and barriers that older adults encounter in using PA trackers	Six women aged 67-78 years who had no prior experience using wearable activity trackers but owned a smartphone. Intentional convenience sampling technique	A focus group was conducted.	Three themes assessed the barriers, facilitators, and areas for improvement perceived by the participants in the use of the activity monitoring devices. Barriers: problems encountered when using the devices caused them anxiety, and they had to ask for help or call the researcher. In addition, they felt that the instructions provided were very brief and not in their native language. In addition, the devices were not waterproof, were incompatible or difficult to synchronize with some smartphones, there was inaccuracy in the step count, and they did not measure other clinical parameters (blood pressure, heart rate, etc.). Facilitators: the devices encouraged them to be more active and contributed to their personal well-being. Some participants changed their daily behavior due to the use of the device, comparing themselves with each other based on the steps they were able to take, or the information provided by their trackers. Areas for improvement: participants did not perceive that the feedback provided by the devices forced them to be more active.	Older adults are motivated to use PA monitoring devices. However, they perceive some problematic aspects that may cause some rejection in their use, such as the instructions provided, the handling, or the synchronization of these devices with the smartphone. These aspects need to be considered for adequate use of PA monitoring in this age group.	10/10
Batsis et al (2016) [40]	Phenomenology	To evaluate the reliability and acceptability of a PA monitoring device (Fitbit) to promote behavior change in older adults with obesity residing in rural areas	Eight older adults (4 women and 4 men) aged 65-80 years with BMI greater than 30 kg/m^2^ and in need of weight loss. All participants had smartphones. Intentional convenience sampling technique	A semistructured interview was conducted with each participant	The reliability and acceptability of the PA monitoring device in older adults in rural areas were evaluated and summarized in the following barriers and facilitators: Barriers: it was observed that after the use of the PA monitoring devices, the total physical activity time of older adults decreased. Facilitators: participants would recommend the use of the device as they were easy to use and found the feedback provided by the device useful. In addition, the social interaction was positively valued and motivated them to use the device, causing a positive behavior change. Finally, participants stated that the results obtained by the device should be explained by a health professional, trainer, and so on	Although the use of Fitbit is accepted by older adults with obesity, they perceive that they need advice from people (health care professionals, trainers, and so on) to teach them how to interpret the results obtained on the device and increase their confidence in the use of the device, thus bringing about a change in their behavior.	10/10

a PA: physical activity.

b mHealth: mobile health.

All the studies analyzed included adults older than 50 years, whose ages ranged broadly from 51 to 94 years. Most included mixed-gender samples, although one study exclusively analyzed the perspective of women [39]. With respect to comorbidities and functional status, several studies have moved beyond healthy populations to include individuals with chronic illnesses, older adults with obesity (BMI >30 kg/m^2^), and participants with mild walking disabilities using wheeled walkers. Notably, formal categorizations of socioeconomic status (SES), culture, and ethnicity were absent from the primary studies’ demographic reports. In terms of geographic scope, 5 studies were conducted in the United States [32-34,36,40], 2 in Canada [37,38], and the remaining in Sweden [35], Ireland [31], and Germany [39].

Concerning the objectives, 2 studies analyzed the barriers and facilitators perceived by older adults regarding the use of PA monitoring devices [33,39], another analyzed the experience of this population group using PA monitoring devices and their perceptions of factors influencing their long-term use [32], and the remaining analyzed the experiences, acceptability, effects, and preferences of older adults regarding the use of PA monitoring devices [34-38,40].

Most studies used the phenomenological approach [32-40], except for one study that used meta-ethnography [31]. The main data collection techniques used were focus groups [33,37], semistructured interviews [32,34,36,38,40], or a combination of both [31,35,39].

### Description of the Main Findings

Seven of the analyzed studies reported the barriers, facilitators, and areas of improvement perceived by adults for the use of wearable mHealth technologies for PA promotion [31,33-35,37,38,41]; 1 synthesized the facilitators that promoted the use of wearable mHealth technologies among older adults [32], and 2 analyzed the facilitators and barriers that facilitated or hindered the use of these devices and apps [36,40].

Three major themes emerged from the narratives explaining older adults’ perceptions of wearable mHealth technologies promoting PA: (1) barriers to their use of wearable mHealth technologies to promote PA: personal (physical and perceptions about technology and tastes and preferences), technological (functionality, content, design, alarms, availability, and accessibility), and environmental (seasonal variations); (2) facilitators of their use of wearable mHealth technologies to promote PA: personal (consideration that it improves health, perceptions about technology and tastes and preferences), technological (functionality, content, and design), relational (technological support and social support), environmental (seasonal variations), and health sector (support and monitoring of health services); and (3) main areas of improvement for the use of wearable mHealth technologies to promote PA: personal (perceptions about technology, tastes, and preferences), technological (functionality, content, and design), and relational (technological support). These themes are presented below with the corresponding categories, subcategories, and codes (Tables 2–4).

**Table 2.** Perceived barriers to the use of wearable mHealth^a^ technologies for physical activity promotion by older adults (categories, subcategories, and codes).

Categories, subcategories, and codes	References
Personal barriers	
Physical aspects	
Decline in physical health	[31-33,35,37,39]
Physical limitations	[31,32,35]
Slow processing speed	[31,33,34]
Perceptions about technologies	
Lack of skill and insecurity in the use of technology	[31,33-36,38,39]
Low perception of technology use	[31,33-36,38,39]
Lack of previous experience	[31,33,34,38]
Negative feelings	[31,38-40]
Lack of interest and curiosity	[33,34,38]
Making mistakes by not asking for help from others around them	[31,38]
Personal preferences	
Change of routine	[31,33-35,38]
Decline in physical health	[35]
Technological barriers	
Functionality	
Cumbersome design	[31,33-35,37-39]
Technological problems with the devices	[31-35,38,39]
Lack of desired functions	[31,33-35,38,39]
Not age-friendly technology	[31,33-35,37,38]
Difficult to use	[31,33-35,38,39]
Unreliable	[31,33,35,36,39]
Complicated tablet or smartphone	[31,33-35]
Requires a smartphone	[31,34]
Not helpful in troubleshooting	[39]
Poor, absent, or negative feedback from the device	[31]
Content	
Not capturing all activities	[31,33,35]
Automatic targeting function	[31]
Design	
Large and rigid band	[31,33,35,38]
Frequent loading	[31,34,38]
Privacy	[33,35,38]
Fragile	[35,37,38]
Uncertainties about water or load damage	[31,35,39]
Difficult to put on	[31,35,36]
Big	[35,38]
Discomfort during nocturnal wear	[31,35]
Looks like a medical device (aesthetics)	[31]
Does not match clothing	[31]
Complicated interaction when on the ankle	[31]
Alarms	
Frequent alarms or notifications	[31,34]
Availability	
High price	[33,34]
Accessibility	
Access to instructions, lack of training, and technicalities	[31,35,37-39]
Lack of practical training	[31,33]
Environmental barriers	
Seasonal variations	
Climatic conditions	[37,40]

a mHealth: mobile health.

**Table 3.** Perceived facilitators to the use of wearable mHealth^a^ technologies for physical activity promotion by older adults (categories, subcategories, and codes).

Categories, subcategories, and codes	References
Personal facilitators	
Believe it improves health	
Improved physical activity	[33,35,39]
Stress reduction	[33,39]
Pain reduction	[33,39]
Improved health and fitness	[31-40]
Perceptions about technology	
Intrinsic motivation	[31-40]
Ability to use the device	[31,33-36,38]
Previous experience using technology	[31,33-35,38]
Confidence in the ability to remember how the technology works	[31,34,35,38]
Higher levels of actual physical activity than perceived by the technology	[31,33-40]
Personal preferences	
Positive attitude toward technology use	[31,34-40]
Achievement of personal goals	[31,32,35,37,39,40]
Perceived usefulness of technology	[31,33-35,38,39]
Emotional attachment to devices	[31,34,35,39]
Curiosity and interest in technology use	[31,35,38]
Including technology in users’ daily routine	[32,38]
Technological facilitators	
Functionality	
Receive positive, real-time feedback from the device	[31-35,37-40]
Easy to use	[31-35,38,39]
Smart	[31,33,35,37,38]
Large, easy-to-read display	[31,33,37,38]
Automatic activity recording	[31,33,35,38]
Compatible with other daily life activities	[31,35,39]
Easy synchronization	[31,33]
Simple application	[31]
Large, easy-to-press buttons	[31]
Easy to see	[31]
Content	
Health-related features	[31,33-35,37,39]
Customizable notifications or alarms	[31-35,37]
Goal tracking	[31-33,35,38]
Step counting	[31,35,37,38]
Other device features (light and stopwatch)	[31,33,40]
Sleep tracking	[31,33,38]
Global Positioning System	[31]
Help section	[31]
Design	
Comfortable location on the body and comfortable to wear	[31,37,38]
Small battery and longer battery life	[31,33,38]
Thin and flexible band	[31,33,38]
Pleasant or cool appearance	[31,33,38]
Water resistant	[31,33]
Device looks like a watch	[31,33]
Smaller design	[31,35]
Secure attachment	[31]
Relational facilitators	
Technological support	
Instructions and help to solve problems	[31]
Social support	
Support from family and friends	[31,33,34,38-40]
Peer support, interaction, and communication	[31,33,34,39,40]
Environmental facilitators	
Seasonal variations	
Weather conditions	[33,40]
Health care professionals	
Support and follow-up of health professionals	
Being a tool for health care professionals for follow-up and monitoring	[31,36-38,40]
Patient benefits due to the involvement of health care professionals	[31,36-38,40]
Being part of the treatment plan	[31,32,35,37,39]
Visualization of health information	[31,33-35]
Care team support	[31,32,37,40]
Sharing data with health care professionals	[33,35,38]

a mHealth: mobile health.

**Table 4.** Areas of improvement for the use of wearable mHealth^a^ technologies for the promotion of physical activity by older adults (categories, subcategories, and codes).

Categories, subcategories, and codes	References
Personal areas	
Perceptions about technology	
Intrinsic motivation	[36]
Tastes and preferences	
Data visualization	[33-35,40]
Device feedback	[34,39]
Security	[37,38]
Personal goals	[34]
Attractiveness	[38]
Technological areas	
Functionality	
Age-friendly devices	[31,36,37,39]
Device reliability	[31,34]
Intelligibility	[31,38]
Synchronization with the smartphone	[33]
Large screen format and clear reading	[37]
Content	
Include health-related content	[34,35,39]
Monitoring of other health parameters	[34]
Usability	[38]
Design	
Instructions in native language	[35,39]
Comfortable devices	[31,38]
Sufficient battery life	[31,35]
Relational areas	
Technology support	
Simple, nontechnical instructions	[31,33,37]
Previous training	[31]

a mHealth: mobile health.

### Barriers to the Use of Wearable mHealth Technologies for PA Promotion

Through the reciprocal translation of the primary literature, the synthesis demonstrates that mHealth ecosystem adoption among older adults is structurally hindered by an intersection of age-associated physiological decline and inadequate user-centered design. Personal barriers encompass sensory and physical deterioration, slower information processing speeds, and technology-induced anxiety, which frequently collide with an established reluctance to alter daily habits [31-35,37-40]. This vulnerability is severely compounded by technical deficits; users routinely experience frustration regarding cumbersome and fragile hardware, complex smartphone synchronization requirements, a lack of automated troubleshooting, and unreliable data visualization [31-39]. Furthermore, frequent notifications or alarms act as intrusive stressors [31,34], while high purchasing costs [33,34] and jargon-heavy instructions create severe access barriers [31,33,35,37-39]. These technical challenges are further compounded by environmental variables, as adverse seasonal weather (winter, rain, snow, and so on) restricts physical outdoor use [37,40]. These barriers are detailed in Table 2.

### Facilitators for the Use of Wearable mHealth Technologies for PA Promotion

Conversely, a positive line of argument emerges when wearable technologies successfully bridge the gap between intrinsic health motivation and intuitive functionality and desire for functional independence [31-40]. At the individual level, older adults demonstrate high adherence when they perceive that tracking tools directly translate into stress reduction, pain management, and measurable physical wellness [32-40]. From a technological standpoint, sustained engagement is facilitated by seamless, unobtrusive hardware configurations—specifically watch-like, waterproof devices featuring long battery life, flexible bands, and large, legible displays [31-35,37-40]. Software features that offer automated tracking, personalized goals, gamified self-competition, and real-time positive feedback act as powerful behavioral catalysts [31-35,37-40]. Critically, the synthesis highlights that hardware and software fail in isolation; relational and systemic scaffolding is paramount. Sustained use is driven by robust social networks (family and peers) [31,33,34,38-40] and, fundamentally, by the formal involvement of health care professionals who integrate these digital metrics into active clinical treatment plans and continuous medical monitoring [31-33,35-38,40]. Table 3 maps the comprehensive structure of these facilitators.

### Areas of Improvement for the Use of Wearable mHealth Technologies for PA Promotion

To minimize early abandonment and maximize clinical utility, the synthesized data establishes explicit pathways for age-inclusive development. Participants universally demand a shift toward age-friendly engineering that prioritizes intuitive interfaces, native-language instruction manuals, enhanced data privacy, and the expansion of monitored metrics to include broader clinical parameters beyond basic pedometry [31,33-39]. Relatably, older adults highlight that closing the digital divide requires robust instructional scaffolding. Developers and health care networks must cooperate to provide accessible, nontechnical training and ongoing troubleshooting support to foster digital self-efficacy and prevent technology rejection [31,33,37]. These strategic directions for technological and clinical adaptation are synthesized in Table 4.

### Results of the Analysis of the Quality of the Studies Analyzed

The methodological quality of the articles was analyzed with the JBI-QARI [30]. Table 5 shows the detailed results of the analysis of the methodological quality of the included studies after this tool was applied.

**Table 5.** Analysis of the quality of the studies analyzed with the Joanna Briggs Institute Qualitative Assessment and Review Instrument.

Author/s	Is there congruity between the stated philosophical perspective and the research methodology?	Is there congruity between the research methodology and the research question or objectives?	Is there congruity between the research methodology and the methods used to collect data?	Is there congruity between the research methodology and the representation and analysis of data?	Is there congruity between the research methodology and the interpretation of results?	Is there a statement locating the researcher culturally or theoretically?	Is the influence of the researcher on the research, and vice-versa, addressed?	Are participants, and their voices, adequately represented?	Is the research ethical according to current criteria or, for recent studies, and is there evidence of ethical approval by an appropriate body?	Do the conclusions drawn in the research report flow from the analysis, or interpretation, of the data?	Total
Moore et al 2021 [31]	Yes	Yes	Yes	Yes	Yes	Yes	Yes	Yes	Yes	Yes	10/10
Peng et al 2021 [32]	Yes	Yes	Yes	Yes	Yes	Yes	Yes	Yes	Yes	Yes	10/10
Kononova et al 2019 [33]	Yes	Yes	Yes	Yes	Yes	Yes	Yes	Yes	Yes	Yes	10/10
Abouzahra and Ghasemaghaei 2020 [34]	Yes	Yes	Yes	Yes	Yes	Yes	Yes	Yes	Yes	Yes	10/10
Ehn et al 2018 [35]	Yes	Yes	Yes	Yes	Yes	Yes	Yes	Yes	Yes	Yes	10/10
Fausset et al 2013 [36]	Yes	Yes	Yes	Yes	Yes	Yes	Yes	Yes	Yes	Yes	10/10
Mercer et al 2016 [37]	Yes	Yes	Yes	Yes	Yes	Yes	Yes	Yes	Yes	Yes	10/10
Puri et al 2017 [38]	Yes	Yes	Yes	Yes	Yes	Yes	Yes	Yes	Yes	Yes	10/10
Schlomann et al 2016 [39]	Yes	Yes	Yes	Yes	Yes	Yes	Yes	Yes	Yes	Yes	10/10
Batsis et al 2016 [40]	Yes	Yes	Yes	Yes	Yes	Yes	Yes	Yes	Yes	Yes	10/10

## Discussion

### Principal Results

The objective of this qualitative systematic review was to analyze the point of view of older adults on the aspects that can influence the use of wearable mHealth technologies for the promotion of PA and to analyze their demands, contexts, and desires to develop more accessible, age-friendly, and acceptable technologies for this population group [19].

People aged 50 years and older perceive personal barriers (physical aspects, perceptions about technologies, and tastes or preferences), technological barriers (functionality, content, design, alarms, availability, and accessibility), and environmental barriers (seasonal variations) that hinder the use of wearable mHealth technologies for the promotion of PA [31-38,40,41]. On the other hand, this population includes personal facilitators (which consider that it improves health, perceptions about technologies, and tastes or preferences), technological facilitators (functionality, content, and design), relational facilitators (technological support and social support), environmental facilitators (seasonal variations), and health professionals (support and follow-up by health professionals) that favor the use of wearable mHealth technologies [31-38,40,41]. Likewise, people more than 50 years of age perceive personal factors (perceptions about technologies and tastes or preferences), technological factors (functionality, content, and design), and relational factors (technological support) as areas for improvement in mHealth devices and apps for the promotion of PA [31,33-38,40,41].

To illustrate the practical relevance and tangible impact of these technologies, specific mHealth interventions from synthesized studies demonstrate how targeted features can drive positive behavioral changes in vulnerable cohorts. For instance, the implementation of a commercial wearable device (Fitbit) among older adults with obesity residing in rural areas proved highly acceptable; it successfully motivated positive behavior change by providing useful real-time feedback and fostering social interaction [40]. Similarly, in a cohort of older adults living with chronic illnesses, wearable activity trackers functioned as powerful behavioral catalysts [37]. By using alert functions and setting daily step goals, these devices gamify PAs, significantly increasing users’ awareness of their daily movement and transforming exercise into self-competition [37]. Furthermore, devices that allowed users to set small, progressive goals, supported by reminders and structured routines—such as wearing the device first thing in the morning—were instrumental in helping older adults develop long-term PA habits [32]. The increased self-awareness facilitated by these trackers also motivated older adults to make broader lifestyle changes and proactively seek health-related information [38]. These empirical examples underscore that when wearable mHealth technologies successfully integrate user-friendly feedback, gamification, and social support, they effectively translate theoretical facilitators into measurable health impacts for aging populations [32,37,38,40].

### Comparison With Prior Work

In line with previous studies that have analyzed the use of wearable mHealth technologies among older adults [4,42], our results confirm that the following barriers hinder their use: physiological state, age, vision loss, hearing loss [31], high price [33,34], lack of interest in older adults, and inexperience and fear of using technologies and sharing data [33-35,37,38]. In addition, the results of this review coincide with those of previous studies showing the following facilitators for the use of devices and apps among older adults: previous experience with technology [32,34,38], being user-friendly [31,35], and favoring the independence and autonomy of older adults [33,35,38].

Another previous study performed on people with low back pain [43] confirmed, as in our review, that older adults must be supported by family members, friends, health professionals, and other users to be able to continuously use PA monitoring devices and apps [31,33,34,37,39,40]. In addition, older adults positively value the use of reminders and alarms [32-35,37] and receive feedback on whether they are using the device correctly [38,40]. Our results are in line with those of previous studies analyzing the use of mHealth ecosystems to promote PA in patients with myeloid neoplasms [25], revealing the need to establish specific objectives according to physiological conditions so that users can use PA monitoring devices appropriately [31,32,35].

In contrast to our findings [31,33,38,39], a study involving adolescent and young adult cancer survivors reported that PA monitoring devices were easy to use and synchronize, allowing for quick data retrieval [44]. Such ease of use is not reflected in the narratives of older adults in our review. Instead, older participants frequently expressed the belief that these technologies were not designed for them [31,35,39]. Moreover, in contrast to studies that claim that devices and apps are affordable and economically profitable [33,34], our results show that cost and access to mHealth technology are barriers for older adults [31,33-35,37,38,41]. These results highlight the need for mHealth technology to be friendlier to older adults, which has been termed age-friendly*,* and to be more flexible to potential users. One study reported that mHealth devices and apps should contain simple functions and simple navigation instructions to reduce the cognitive load of older adults, common and simple language, the option of zoom or large print text, and voice navigation (if they are visually impaired) [45]. These findings are consistent with the results of our studies [31-35,37-39].

For these reasons, governments, international organizations, health professionals, academic institutions, media, and the private sector must consider all these findings to eliminate the existing digital gap between older adults and the use of mHealth devices and apps. In this way, through learning and training, older adults can benefit from the use of wearable mHealth technologies by achieving better self-management of their health [46].

However, advocating for inclusive design is insufficient without addressing the economic realities of the digital health ecosystem. The technology sector generally recognizes the value of user-centered interfaces; rather, the primary barrier is economic. The high costs and lower profit margins associated with developing customized platforms often fail to support a compelling business case. To genuinely drive industry change, future research must actively develop this business case. By leveraging resources largely inaccessible to private developers—such as longitudinal electronic health records, clinical registries, and biobanks—the academic community must conduct robust Health Economics and Outcomes Research and budget impact analyses. Demonstrating a clear clinical and economic return on investment is essential for incentivizing the tech sector to prioritize and fund highly customizable, age-friendly mHealth technologies.

### Limitations

This review included only articles in English and Spanish in the selected databases and excluded relevant articles on the phenomenon studied. In addition, no articles have studied the long-term use of wearable mHealth technologies among older adults, nor have the views of health professionals been considered. On the other hand, our study was based solely on the use of mHealth ecosystems that promote PA, and future studies should continue to investigate the repercussions of these devices and apps over time and from the point of view of professionals.

Another limitation of this meta-ethnography is the restricted demographic reporting in the primary literature. Although some studies detailed clinical parameters and geographic contexts, critical sociodemographic determinants such as SES, culture, and ethnicity were systematically omitted. This paucity of granular data precludes a robust intersectional analysis, obscuring how overlapping social identities and structural inequalities influence mHealth engagement. Consequently, the current evidence may disproportionately reflect privileged cohorts and fail to capture the unique barriers faced by marginalized subpopulations. To advance health equity, future research must rigorously report diverse, intersectional demographic variables, ensuring that mHealth interventions mitigate rather than exacerbate the digital divide among aging populations.

### Conclusions

This meta-ethnography demonstrates that older adults’ engagement with wearable mHealth technologies for PA is not solely dictated by technological proficiency but by a complex interplay of personal, technological, and environmental factors. While usability constraints, age-related physical limitations, and privacy concerns significantly hinder adoption, older adults clearly recognize the potential of these digital health tools to enhance their well-being. Evidence from the synthesized cohorts highlights that when mHealth interventions use user-centered, age-friendly designs and integrate continuous support from health care professionals, they effectively drive sustained behavioral change and long-term adherence. However, a critical methodological gap persists in the current literature: the systemic omission of granular demographic data, specifically SES, cultural background, and ethnicity. This lack of intersectional reporting precludes a comprehensive understanding of how compounding structural inequalities shape digital health access. If mHealth is to promote true health equity, future research must transition from homogeneous sampling to rigorous, inclusive methodologies. Tech developers and clinicians must collaboratively address the multifaceted needs of diverse, marginalized older adult subpopulations. Only through this intersectional approach can we ensure that the proliferation of wearable mHealth technologies bridges, rather than inadvertently exacerbates, the existing digital divide.

## Supplementary material

10.2196/67157 Multimedia Appendix 1 Search strategy.

10.2196/67157 Multimedia Appendix 2 Examples of first-order, second-order, and third-order constructs.

10.2196/67157 Checklist 1 PRISMA 2020 checklist.

## Abbreviations

ENTREQ: Enhancing Transparency in Reporting the Synthesis of Qualitative Research
JBI-QARI: Joanna Briggs Institute Qualitative Assessment and Review Instrument
mHealth: mobile health
PA: physical activity
PRISMA: Preferred Reporting Items for Systematic Reviews and Meta-Analyses
SES: socioeconomic status
WHO: World Health Organization
