# Correction: Perceptions of Adults Aged 50 Years and Older Regarding the Use of Wearable mHealth Technologies to Promote Physical Activity: Systematic Review and Meta-Ethnography

**DOI:** 10.2196/111082

**Published:** 2026-10-08

**Authors:** Beatriz Rodríguez-Martín, Diego González-Arroyo, Susana Priego-Jiménez, María Lopez-González, María Medrano-Echeverría, Marta Carolina Ruiz-Grao, Celia Álvarez-Bueno

**Affiliations:** 1 Department of Nursing, Faculty of Health Sciences, Physiotherapy and Occupational Therapy, University of Castilla-La Mancha, Talavera de la Reina, Spain; 2 ABC-Age Research Group, University of Castilla-La Mancha, Cuenca, Spain; 3 Network for Research on Chronicity, Primary Care, and Health Promotion (RICAPPS), Barcelona, Spain; 4 University Hospital Nuestra Señora del Prado, Health Service of Castilla-La Mancha, Talavera de la Reina, Spain; 5 University Hospital of Cuenca, Camino de El Terminillo s/n, Cuenca, 16003, Spain, 34 969 17 99 00; 6 Department of Health Sciences, Institute for Innovation & Sustainable Food Chain Development, Public University of Navarra, Pamplona, Spain; 7 CIBER de Fisiopatología de la Obesidad y Nutrición (CIBEROBN), Instituto de Salud Carlos III, Madrid, Spain; 8 Department of Nursing, Nursing Faculty, Physiotherapy and Occupational Therapy, University of Castilla-La Mancha. Albacete, Albacete, Spain; 9 Universidad Politécnica y Artística del Paraguay, Asuncion, Paraguay

In “Perceptions of Adults Aged 50 Years and Older Regarding the Use of Wearable mHealth Technologies to Promote Physical Activity: Systematic Review and Meta-Ethnography” [1], the authors noted one error.

The name of the sixth author in the citation information has changed from the following:

Carolina Ruiz-Grao M

The name in the citation information now reads:

Ruiz-Grao MC

The correction will appear in the online version of the paper on the JMIR Publications website together with the publication of this correction notice. Because this was made after submission to PubMed, PubMed Central, and other full-text repositories, the corrected article has also been resubmitted to those repositories.
