# Supplementary material for: Perceptions of Adults Aged 50 Years and Older Regarding the Use of Wearable mHealth Technologies to Promote Physical Activity: Systematic Review and Meta-Ethnography
Source: JMIR Mhealth Uhealth. 2026 Jul 29;14:e67157. doi: 10.2196/67157 (PMC13420493; doi:10.2196/67157)
Supplement: Multimedia Appendix 1 [file mhealth-v14-e67157-s001.docx]

**Multimedia Appendix 1. Search strategy used in each of the databases.**

| MEDLINE (Pubmed) | (("telemedicine"[MeSH Terms] OR "telemedicine"[All Fields] OR ("mobile"[All Fields] AND "health"[All Fields]) OR "mobile health"[All Fields]) AND (("older"[All Fields] OR "olders"[All Fields]) AND ("people s"[All Fields] OR "peopled"[All Fields] OR "peopling"[All Fields] OR "persons"[MeSH Terms] OR "persons"[All Fields] OR "people"[All Fields] OR "peoples"[All Fields])) AND ("exercise"[MeSH Terms] OR "exercise"[All Fields] OR ("physical"[All Fields] AND "activity"[All Fields]) OR "physical activity"[All Fields]) AND ("qualitative research"[MeSH Terms] OR ("qualitative"[All Fields] AND "research"[All Fields]) OR "qualitative research"[All Fields])) AND ((ffrft[Filter]) AND (english[Filter] OR spanish[Filter]) AND (aged[Filter]) AND (2013:2023[pdat])) |
| --- | --- |
| Scopus | Mobile health AND physical activity AND older people AND qualitative research AND PUBYEAR > 2012 AND PUBYEAR < 2024 AND (LIMIT-TO (SUBJAREA,"MEDI" ) ) AND (LIMIT-TO (DOCTYPE,"ar" ) ) AND (LIMIT-TO (LANGUAGE,"Spanish" ) OR LIMIT-TO (LANGUAGE,"English" ) ) AND (LIMIT-TO (EXACTKEYWORD,"Telemedicine" ) OR LIMIT-TO (EXACTKEYWORD,"Qualitative Research" ) OR LIMIT-TO (EXACTKEYWORD,"Mobile Application" ) OR LIMIT-TO (EXACTKEYWORD,"MHealth" ) OR LIMIT-TO (EXACTKEYWORD,"Mobile Applications" ) OR LIMIT-TO (EXACTKEYWORD,"Older Adults" ) OR LIMIT-TO (EXACTKEYWORD,"Systematic Review" ) OR LIMIT-TO (EXACTKEYWORD,"Semi Structured Interview" ) OR LIMIT-TO (EXACTKEYWORD,"Mobile Health" ) OR LIMIT-TO (EXACTKEYWORD,"Physical Activity" ) OR LIMIT-TO (EXACTKEYWORD,"Mobile Phone" ) OR LIMIT-TO (EXACTKEYWORD,"Aged" ) OR LIMIT-TO (EXACTKEYWORD,"Smartphone" ) OR LIMIT-TO (EXACTKEYWORD,"Usability" ) OR LIMIT-TO (EXACTKEYWORD,"Telehealth" ) OR LIMIT-TO (EXACTKEYWORD,"Aging" ) OR LIMIT-TO (EXACTKEYWORD,"Mobile App" ) OR LIMIT-TO (EXACTKEYWORD,"Motivation" ) OR LIMIT-TO (EXACTKEYWORD,"Wearable Technology" ) OR LIMIT-TO (EXACTKEYWORD,"Questionnaire" ) OR LIMIT-TO (EXACTKEYWORD,"Qualitative" ) OR LIMIT-TO (EXACTKEYWORD,"Surveys And Questionnaires" ) OR LIMIT-TO (EXACTKEYWORD,"Interview" ) OR LIMIT-TO (EXACTKEYWORD,"Older People" ) OR LIMIT-TO (EXACTKEYWORD,"Focus Groups" ) OR LIMIT-TO (EXACTKEYWORD,"Feasibility" ) OR LIMIT-TO (EXACTKEYWORD,"Focus Group" ) OR LIMIT-TO (EXACTKEYWORD,"Activity Tracker" ) OR EXCLUDE (EXACTKEYWORD,"Australia" ) OR EXCLUDE (EXACTKEYWORD,"Wellbeing" ) OR EXCLUDE (EXACTKEYWORD,"Prospective Study" ) OR EXCLUDE (EXACTKEYWORD,"China" ) OR EXCLUDE (EXACTKEYWORD,"Research Design" ) OR EXCLUDE (EXACTKEYWORD,"Healthy Lifestyle" ) OR EXCLUDE (EXACTKEYWORD,"Cross-Sectional Studies" ) OR EXCLUDE (EXACTKEYWORD,"Clinical Trial" ) OR EXCLUDE (EXACTKEYWORD,"Cost Effectiveness Analysis" ) OR EXCLUDE (EXACTKEYWORD,"Health Service" ) OR EXCLUDE (EXACTKEYWORD,"Glucose Blood Level" ) OR EXCLUDE (EXACTKEYWORD,"Age" ) OR EXCLUDE (EXACTKEYWORD,"Multicenter Study" ) OR EXCLUDE (EXACTKEYWORD,"Intervention" ) OR EXCLUDE (EXACTKEYWORD,"Fatigue" ) OR EXCLUDE (EXACTKEYWORD,"Self Report" ) OR EXCLUDE (EXACTKEYWORD,"Physiology" ) OR EXCLUDE (EXACTKEYWORD,"Patient Participation" ) OR EXCLUDE (EXACTKEYWORD,"Content Analysis" ) OR EXCLUDE (EXACTKEYWORD,"User-centered Design" ) OR EXCLUDE (EXACTKEYWORD,"Primary Medical Care" ) OR EXCLUDE (EXACTKEYWORD,"Cohort Analysis" ) OR EXCLUDE (EXACTKEYWORD,"Clinical Outcome" ) OR EXCLUDE (EXACTKEYWORD,"Cardiovascular Diseases" ) OR EXCLUDE (EXACTKEYWORD,"Nutrition" ) OR EXCLUDE (EXACTKEYWORD,"Epidemiology" ) OR EXCLUDE (EXACTKEYWORD,"Intervention Study" ) OR EXCLUDE (EXACTKEYWORD,"Cerebrovascular Accident" ) OR EXCLUDE (EXACTKEYWORD,"Home Care" ) OR EXCLUDE (EXACTKEYWORD,"Controlled Study" ) OR EXCLUDE (EXACTKEYWORD,"Middle Aged" ) OR EXCLUDE (EXACTKEYWORD,"Procedures" ) OR EXCLUDE (EXACTKEYWORD,"Self Care" ) OR EXCLUDE (EXACTKEYWORD,"Quality Of Life" ) OR EXCLUDE (EXACTKEYWORD,"Clinical Article" ) OR EXCLUDE (EXACTKEYWORD,"Randomized Controlled Trial" ) OR EXCLUDE (EXACTKEYWORD,"Psychology" ) OR EXCLUDE (EXACTKEYWORD,"Human Experiment" ) OR EXCLUDE (EXACTKEYWORD,"Self-management" ) OR EXCLUDE (EXACTKEYWORD,"Major Clinical Study" ) OR EXCLUDE (EXACTKEYWORD,"EHealth" ) OR EXCLUDE (EXACTKEYWORD,"Health Promotion" ) OR EXCLUDE (EXACTKEYWORD,"Internet" ) OR EXCLUDE (EXACTKEYWORD,"Young Adult" ) OR EXCLUDE (EXACTKEYWORD,"Thematic Analysis" ) OR EXCLUDE (EXACTKEYWORD,"Pilot Study" ) OR EXCLUDE (EXACTKEYWORD,"Mental Health" ) OR EXCLUDE (EXACTKEYWORD,"Social Support" ) OR EXCLUDE (EXACTKEYWORD,"Self-Management" ) OR EXCLUDE (EXACTKEYWORD,"Behavior Change" ) OR EXCLUDE (EXACTKEYWORD,"Health Care Personnel" ) OR EXCLUDE (EXACTKEYWORD,"Randomized Controlled Trial (topic)" ) OR EXCLUDE (EXACTKEYWORD,"Information Processing" ) OR EXCLUDE (EXACTKEYWORD,"Outcome Assessment" ) OR EXCLUDE (EXACTKEYWORD,"Kinesiotherapy" ) OR EXCLUDE (EXACTKEYWORD,"Social Media" ) OR EXCLUDE (EXACTKEYWORD,"Telerehabilitation" ) OR EXCLUDE (EXACTKEYWORD,"COVID-19" ) OR EXCLUDE (EXACTKEYWORD,"Caregiver" ) OR EXCLUDE (EXACTKEYWORD,"Non Insulin Dependent Diabetes Mellitus" ) OR EXCLUDE (EXACTKEYWORD,"Self Concept" ) OR EXCLUDE (EXACTKEYWORD,"Adolescent" ) OR EXCLUDE (EXACTKEYWORD,"Rehabilitation" ) OR EXCLUDE (EXACTKEYWORD,"Health Care Delivery" ) OR EXCLUDE (EXACTKEYWORD,"Diabetes Mellitus, Type 2" ) OR EXCLUDE (EXACTKEYWORD,"Pilot Projects" ) OR EXCLUDE (EXACTKEYWORD,"Depression" ) OR EXCLUDE (EXACTKEYWORD,"Medline" ) OR EXCLUDE (EXACTKEYWORD,"Dementia" ) OR EXCLUDE (EXACTKEYWORD,"Anxiety" ) OR EXCLUDE (EXACTKEYWORD,"Caregivers" ) OR EXCLUDE (EXACTKEYWORD,"Text Messaging" ) OR EXCLUDE (EXACTKEYWORD,"Pandemic" ) OR EXCLUDE (EXACTKEYWORD,"Obesity" ) OR EXCLUDE (EXACTKEYWORD,"Health" ) OR EXCLUDE (EXACTKEYWORD,"Risk Factor" ) OR EXCLUDE (EXACTKEYWORD,"Daily Life Activity" ) OR EXCLUDE (EXACTKEYWORD,"Public Health" ) OR EXCLUDE (EXACTKEYWORD,"Patient Education" ) OR EXCLUDE (EXACTKEYWORD,"Patient Compliance" ) OR EXCLUDE (EXACTKEYWORD,"Follow Up" ) OR EXCLUDE (EXACTKEYWORD,"Education" ) OR EXCLUDE (EXACTKEYWORD,"Priority Journal" ) OR EXCLUDE (EXACTKEYWORD,"Lifestyle" ) OR EXCLUDE (EXACTKEYWORD,"Diabetes Mellitus" ) OR EXCLUDE (EXACTKEYWORD,"Health Education" ) OR EXCLUDE (EXACTKEYWORD,"Practice Guideline" ) OR EXCLUDE (EXACTKEYWORD,"Patient Care" ) OR EXCLUDE (EXACTKEYWORD,"Pandemics" ) OR EXCLUDE (EXACTKEYWORD,"Type 2 Diabetes" ) OR EXCLUDE (EXACTKEYWORD,"Independent Living" ) OR EXCLUDE (EXACTKEYWORD,"Health Care" ) OR EXCLUDE (EXACTKEYWORD,"Diabetes" ) OR EXCLUDE (EXACTKEYWORD,"Adolescents" ) OR EXCLUDE (EXACTKEYWORD,"Aging In Place" ) OR EXCLUDE (EXACTKEYWORD,"Algorithm" ) OR EXCLUDE (EXACTKEYWORD,"Arrhythmias" ) OR EXCLUDE (EXACTKEYWORD,"Application" ) OR EXCLUDE (EXACTKEYWORD,"Assessment" ) OR EXCLUDE (EXACTKEYWORD,"Cardiovascular Disease" ) OR EXCLUDE (EXACTKEYWORD,"Cell Phone" ) OR EXCLUDE (EXACTKEYWORD,"Chatbot" ) OR EXCLUDE (EXACTKEYWORD,"Children" ) OR EXCLUDE (EXACTKEYWORD,"Cognitive Defect" ) OR EXCLUDE (EXACTKEYWORD,"Development" ) OR EXCLUDE (EXACTKEYWORD,"Ehealth" ) OR EXCLUDE (EXACTKEYWORD,"Electronic Health Record" ) OR EXCLUDE (EXACTKEYWORD,"Game Elements" ) OR EXCLUDE (EXACTKEYWORD,"HIV" ) OR EXCLUDE (EXACTKEYWORD,"Health Care Costs" ) OR EXCLUDE (EXACTKEYWORD,"Health Coaching" ) OR EXCLUDE (EXACTKEYWORD,"Heart Disease" ) OR EXCLUDE (EXACTKEYWORD,"Atrial Fibrillation" ) OR EXCLUDE (EXACTKEYWORD,"Cardiac Rehabilitation" ) OR EXCLUDE (EXACTKEYWORD,"Child" ) OR EXCLUDE (EXACTKEYWORD,"Child Parent Relation" ) OR EXCLUDE (EXACTKEYWORD,"Cognitive Impairment" ) OR EXCLUDE (EXACTKEYWORD,"Databases, Factual" ) OR EXCLUDE (EXACTKEYWORD,"Family" ) OR EXCLUDE (EXACTKEYWORD,"Family Caregivers" ) OR EXCLUDE (EXACTKEYWORD,"Geriatrics" ) OR EXCLUDE (EXACTKEYWORD,"Parents" ) OR EXCLUDE (EXACTKEYWORD,"Occupational Therapy" ) OR EXCLUDE (EXACTKEYWORD,"Primary Health Care" ) OR EXCLUDE (EXACTKEYWORD,"Privacy" ) OR EXCLUDE (EXACTKEYWORD,"Social Isolation" ) OR EXCLUDE (EXACTKEYWORD,"Gamification" ) OR EXCLUDE (EXACTKEYWORD,"Healthcare" ) OR EXCLUDE (EXACTKEYWORD,"Human Factors" ) OR EXCLUDE (EXACTKEYWORD,"Loneliness" ) OR EXCLUDE (EXACTKEYWORD,"Self-care" ) OR EXCLUDE (EXACTKEYWORD,"Sleep" ) OR EXCLUDE (EXACTKEYWORD,"Wheelchair" ) OR EXCLUDE (EXACTKEYWORD,"Male" ) OR EXCLUDE (EXACTKEYWORD,"Female" ) OR EXCLUDE (EXACTKEYWORD,"Ecological Momentary Assessment" ) OR EXCLUDE (EXACTKEYWORD,": Sedentary Behavior" ) OR EXCLUDE (EXACTKEYWORD,"Etudes Interlinguistiques" ) OR EXCLUDE (EXACTKEYWORD,"AI" ) OR EXCLUDE (EXACTKEYWORD,"AIoT" ) OR EXCLUDE (EXACTKEYWORD,"Access To Car" ) OR EXCLUDE (EXACTKEYWORD,"Arrhythmia" ) OR EXCLUDE (EXACTKEYWORD,"Arthritis" ) OR EXCLUDE (EXACTKEYWORD,"Architecture" ) OR EXCLUDE (EXACTKEYWORD,"Arthritis, Rheumatoid" ) OR EXCLUDE (EXACTKEYWORD,"Arthroplasty" ) OR EXCLUDE (EXACTKEYWORD,"Asthma" ) OR EXCLUDE (EXACTKEYWORD,"Asthma Control" ) OR EXCLUDE (EXACTKEYWORD,"Asthma Management" ) OR EXCLUDE (EXACTKEYWORD,"Atmosphere" ) OR EXCLUDE (EXACTKEYWORD,"Augmentative And Alternative Communication" ) OR EXCLUDE (EXACTKEYWORD,"Augmentative-and-alternative Communication" ) OR EXCLUDE (EXACTKEYWORD,"Bariatric Surgery" ) OR EXCLUDE (EXACTKEYWORD,"Bayes Theorem" ) OR EXCLUDE (EXACTKEYWORD,"Becoming Alone") OR EXCLUDE (EXACTKEYWORD,"Behavior") OR EXCLUDE (EXACTKEYWORD,"Behavior Change Intervention") OR EXCLUDE (EXACTKEYWORD,"COVID-19 Pandemic" ) OR EXCLUDE (EXACTKEYWORD,"Blended Health Intervention") OR EXCLUDE (EXACTKEYWORD,"Biofeedback") OR EXCLUDE (EXACTKEYWORD,"Biobehavioral Sciences) OR EXCLUDE (EXACTKEYWORD,"Big Data" ) OR EXCLUDE (EXACTKEYWORD,"Behavior" ) OR EXCLUDE (EXACTKEYWORD,"Cancer") OR EXCLUDE (EXACTKEYWORD,"Physiotherapy") OR EXCLUDE (EXACTKEYWORD,"Frailty") OR EXCLUDE (EXACTKEYWORD,"Physicians") OR EXCLUDE (EXACTKEYWORD,"Osteoarthritis" ) OR EXCLUDE (EXACTKEYWORD,"Medical Informatics") OR EXCLUDE (EXACTKEYWORD,"Intellectual Disability" ) OR EXCLUDE (EXACTKEYWORD,"Health Literacy" ) OR EXCLUDE (EXACTKEYWORD,"Fall Risk" ) OR EXCLUDE (EXACTKEYWORD,"COPD" ) OR EXCLUDE (EXACTKEYWORD,"Eating" ) OR EXCLUDE (EXACTKEYWORD,"EQ-5D-5 L Questionnaire") OR EXCLUDE (EXACTKEYWORD,"Drug Use" ) OR EXCLUDE (EXACTKEYWORD,"Drug Overdose") OR EXCLUDE (EXACTKEYWORD,"Drug Monitoring") OR EXCLUDE (EXACTKEYWORD,"Case Study" ) OR EXCLUDE (EXACTKEYWORD,"CeHRes Roadmap" ) OR EXCLUDE (EXACTKEYWORD,"Charles Bonnet Syndrome) OR EXCLUDE (EXACTKEYWORD,"Childhood Cancer" ) OR EXCLUDE (EXACTKEYWORD,"Chondroitin Sulfate") OR EXCLUDE (EXACTKEYWORD,"Chronic Obstructive Lung Disease") OR EXCLUDE (EXACTKEYWORD,"Colorectal Cancer") OR EXCLUDE (EXACTKEYWORD,"Comprehensive Geriatric Assessment" ) OR EXCLUDE EXACTKEYWORD,"Concept Formation") OR EXCLUDE (EXACTKEYWORD,"Cystic Fibrosis") OR EXCLUDE (EXACTKEYWORD,"Dependency") OR EXCLUDE (EXACTKEYWORD,"Diabetes Mellitus Type 2") OR EXCLUDE (EXACTKEYWORD,"Dialysis" ) OR EXCLUDE (EXACTKEYWORD,"Diet") OR EXCLUDE EXACTKEYWORD,"Dietary Supplement") OR EXCLUDE (EXACTKEYWORD,"Ecological Momentary Assessment (EMA)") OR EXCLUDE (EXACTKEYWORD,"Electrodermal Response") OR EXCLUDE (EXACTKEYWORD,"Environment And Public Health") OR EXCLUDE (EXACTKEYWORD,"Experience Sampling Method") OR EXCLUDE (EXACTKEYWORD,"Fall Prevention") OR EXCLUDE (EXACTKEYWORD,"Falls") OR EXCLUDE (EXACTKEYWORD,"GERIATRIC MEDICINE") OR EXCLUDE (EXACTKEYWORD,"Game Design" ) OR EXCLUDE EXACTKEYWORD,"Gaming Experience") OR EXCLUDE (EXACTKEYWORD,"Geriatric Nursing" ) OR EXCLUDE (EXACTKEYWORD,"Gestational" ) OR EXCLUDE (EXACTKEYWORD,"Gestational Weight Gain") OR EXCLUDE (EXACTKEYWORD,"Graded In" ) OR EXCLUDE (EXACTKEYWORD,"Green Infrastructure" ) OR EXCLUDE (EXACTKEYWORD,"Grounded Theory") OR EXCLUDE (EXACTKEYWORD,"Guidelines" )) |
| Web of Science (WOS) | **(((TS=(Mobile health)) AND TS=(PHYSICAL ACTIVITY)) AND TS=(OLDER PEOPLE)) AND TS=(QUALITATIVE RESEARCH)** |
| ProQuest | (Mobile Health AND physical activity AND older people AND qualitative research) AND (stype.exact("Scholarly Journals") AND la.exact("ENG") AND subt.exact(("aging" OR "adults" OR "qualitative research" OR "physical fitness" OR "systematic review") AND ("physical activity" OR "smartphones" OR "focus groups") AND "older people" NOT ("intervention" OR "geriatrics" OR "caregivers" OR "dementia" OR "covid-19" OR "mental health" OR "health services" OR "social networks" OR "activities of daily living" OR "pandemics" OR "coronaviruses" OR "society" OR "social isolation" OR "design" OR "frailty" OR "alzheimer's disease" OR "loneliness" OR "diabetes" OR "nursing homes" OR "clinical trials" OR "falls" OR "quality of life" OR "behavior" OR "public health" OR "mobility" OR "young adults" OR "neighborhoods" OR "primary care" OR "cross-sectional studies")) |
| The Cochrane Library Plus | mobile health in Title Abstract Keyword AND "physical activity" in Title Abstract Keyword AND older people in Title Abstract Keyword AND "qualitative research" in Title Abstract Keyword |
| CINAHL | mobile health AND physical activity AND older people AND qualitative research |
| ÍnDICEs-CSIC | MHEALTH AND OLDER PEOPLE AND PHYSICAL ACTIVITY AND QUALITATIVE RESEARCH |
