# Supplementary material for: Perceptions of Adults Aged 50 Years and Older Regarding the Use of Wearable mHealth Technologies to Promote Physical Activity: Systematic Review and Meta-Ethnography
Source: JMIR Mhealth Uhealth. 2026 Jul 29;14:e67157. doi: 10.2196/67157 (PMC13420493; doi:10.2196/67157)
Supplement: Multimedia Appendix 2 [file mhealth-v14-e67157-s002.docx]

Multimedia Appendix 2. Examples of first-order, second-order, and third-order constructs.

| **First-order constructs** | **Second-order constructs** | **Third-order constructs** |
| --- | --- | --- |
| “It’s very rigid. The design is poor. It collects water underneath. I end up having a truly loose bracelet… I found it totally uncomfortable. It’s truly ugly” (32) | Band as plastic, clunky, annoying, rigid, and uncomfortable | Theme: Perceived barriers to the use of wearable mHealth technologies for physical activity promotion by older adults.  Category: Technological barriers.  Subcategory: Design.  Code: Large and rigid band.  Theme: Perceived barriers to the use of wearable mHealth technologies for physical activity promotion by older adults.  Category: Technological barriers.  Subcategory: Design.  Code: Cumbersome design. |
| “I thought they were highly inaccurate. I clocked 1,350 steps just driving out to eat in Rapids one day” (32) | What’s Stopping Them: Data Inaccuracy and Lack of Adequate Instructions | Theme: Perceived barriers to the use of wearable mHealth technologies for physical activity promotion by older adults.  Category: Technological barriers.  Subcategory: Functionality.  Code: Unreliable. |
| “I have walked slightly more while being monitored” (38) | Influence on the Individual | Theme: Perceived facilitators to the use of wearable mHealth technologies for physical activity promotion by older adults.  Category: Personal facilitators.  Subcategory: Believe it improves health.  Code: Improved physical activity. |
